# Mortality trends of malignant neoplasm of kidney among 5–85+ year-olds in the United States: a retrospective analysis

**DOI:** 10.3389/fonc.2026.1752889

**Published:** 2026-05-19

**Authors:** Jiafeng Hu, Xueping Sun, Junsheng Jiang

**Affiliations:** 1 Department of Nephrology, The Second Affiliated Hospital of Zhejiang University School of Medicine Linping Campus, Hangzhou, China; 2 Department of Pediatrics, The Second Affiliated Hospital of Zhejiang University School of Medicine Linping Campus, Hangzhou, China

**Keywords:** age adjusted mortality rate, health disparity, malignant neoplasm of kidney, mortality trend, United States

## Abstract

**Background:**

This study aimed to investigate mortality trends associated with malignant neoplasm of the kidney in the United States over a 25-year period (1999–2023) and explore variations across age, sex, race/ethnicity, geographic region, and urbanization level.

**Methods:**

Population-level mortality data were extracted from the Centers for Disease Control and Prevention Wide-ranging Online Data for Epidemiologic Research (CDC WONDER) database. Malignant neoplasm of the kidney was identified using the International Classification of Diseases, 10th Revision (ICD-10) code C64. Age-adjusted mortality rates (AAMRs) per 100,000 population were calculated with direct standardization to the 2000 United States standard population. Joinpoint regression was applied to estimate annual percentage changes (APCs) and average annual percentage changes (AAPCs) with 95% confidence intervals (CIs) to characterize temporal trends.

**Results:**

A total of 10,912 deaths in 1999 and 14,549 deaths in 2023 were attributed to malignant neoplasm of the kidney, representing a 33.33% increase. The overall AAMR decreased significantly from 4.29 per 100,000 (95% CI: 4.21–4.37) in 1999 to 3.61 per 100,000 (95% CI: 3.55–3.67) in 2023, with an AAPC of −0.76 (95% CI: −1.10 to −0.41, P<0.05). Mortality rates increased progressively with advancing age, and the oldest age group (85 years and older) exhibited the highest crude mortality rate, which rose continuously during the study period, whereas most other age subgroups displayed declining CMRs or AAPCs. Males exhibited persistently higher AAMRs than females, and non-Hispanic Black individuals maintained the highest AAMRs among all racial and ethnic groups. Nonmetropolitan (rural) areas demonstrated higher AAMRs than metropolitan (urban) areas throughout the study interval. The Northeast region recorded the most pronounced reduction in AAMR, with an AAPC of −1.55 (P<0.05).

**Conclusion:**

Despite a significant reduction in overall age-adjusted mortality from malignant neoplasm of the kidney in the United States between 1999 and 2023, substantial disparities persist across age, sex, race/ethnicity, geographic region, and urbanization level. Divergent trends between crude mortality counts and age-adjusted rates underscore the dominant influence of population aging on absolute mortality burden.

## 1 Introduction

Renal malignant neoplasms, with renal cell carcinoma as the predominant histological subtype accounting for 80% to 90% of all cases, constitute a major public health challenge in the United States and contribute substantially to national cancer mortality and healthcare utilization (1–3). Widespread improvements in diagnostic imaging, surgical techniques, and systemic therapies have reshaped the clinical care of kidney cancer, yet significant disparities in mortality outcomes across demographic and geographic subgroups remain poorly characterized (4–6).

Although prior epidemiological studies have explored associations between individual sociodemographic factors and kidney cancer mortality, few investigations have conducted a systematic analysis of long-term mortality trends across multiple intersecting determinants including age, sex, race/ethnicity, census region, and urbanization level using nationally representative population data. The CDC WONDER database provides comprehensive, population-based mortality statistics that enable robust assessment of temporal patterns and inequities with high statistical reliability. Using 25 years of consecutive mortality data, this retrospective study aimed to quantify temporal changes in kidney cancer mortality and identify persistent disparities. The findings may inform evidence-based public health planning, resource allocation, and targeted interventions to reduce inequities and alleviate the population burden of kidney cancer.

## 2 Methods

### 2.1 Data source

This retrospective, population-based observational study analyzed mortality data for United States residents aged 5–85+ years from 1999 through 2023, obtained from the CDC WONDER Multiple Cause of Death database. Deaths were included if malignant neoplasm of the kidney (ICD-10 code C64) was documented as an underlying or contributing cause on the death certificate. The study utilized de-identified, publicly available data and was exempt from institutional review board approval. All analyses were performed in compliance with the Strengthening the Reporting of Observational Studies in Epidemiology (STROBE) statement (7–9).

### 2.2 Data extraction

Variables extracted for analysis included year of death, geographic location, sex, age group, race/ethnicity, urban-rural classification, and U.S. Census region. Age was categorized into nine mutually exclusive groups: 5–14, 15–24, 25–34, 35–44, 45–54, 55–64, 65–74, 75–84, and 85 years and older. Race/ethnicity was classified as non-Hispanic White, non-Hispanic Black, non-Hispanic American Indian/Alaska Native, Hispanic/Latino, and non-Hispanic Other, which includes Asian/Pacific Islanders and other unclassified minority groups. Urban-rural status was defined according to the 2013 National Center for Health Statistics (NCHS) classification scheme, distinguishing between metropolitan (urban) and nonmetropolitan (rural) counties. Geographic stratification was based on four U.S. Census regions: Northeast, Midwest, South, and West.

### 2.3 Statistical analysis

Crude mortality rates (CMRs) per 100,000 population were computed for each age group, as age adjustment within age strata is not epidemiologically interpretable. Age-adjusted mortality rates (AAMRs) per 100,000 population were calculated for all other subgroups using direct standardization to the 2000 U.S. standard population. Temporal mortality trends were analyzed using the Joinpoint Regression Program (version 5.0) to estimate APCs and AAPCs with corresponding 95% CIs. A two-sided P-value <0.05 was considered statistically significant.

## 3 Results

### 3.1 Overall mortality trends (1999–2023)

**Figure 1:**
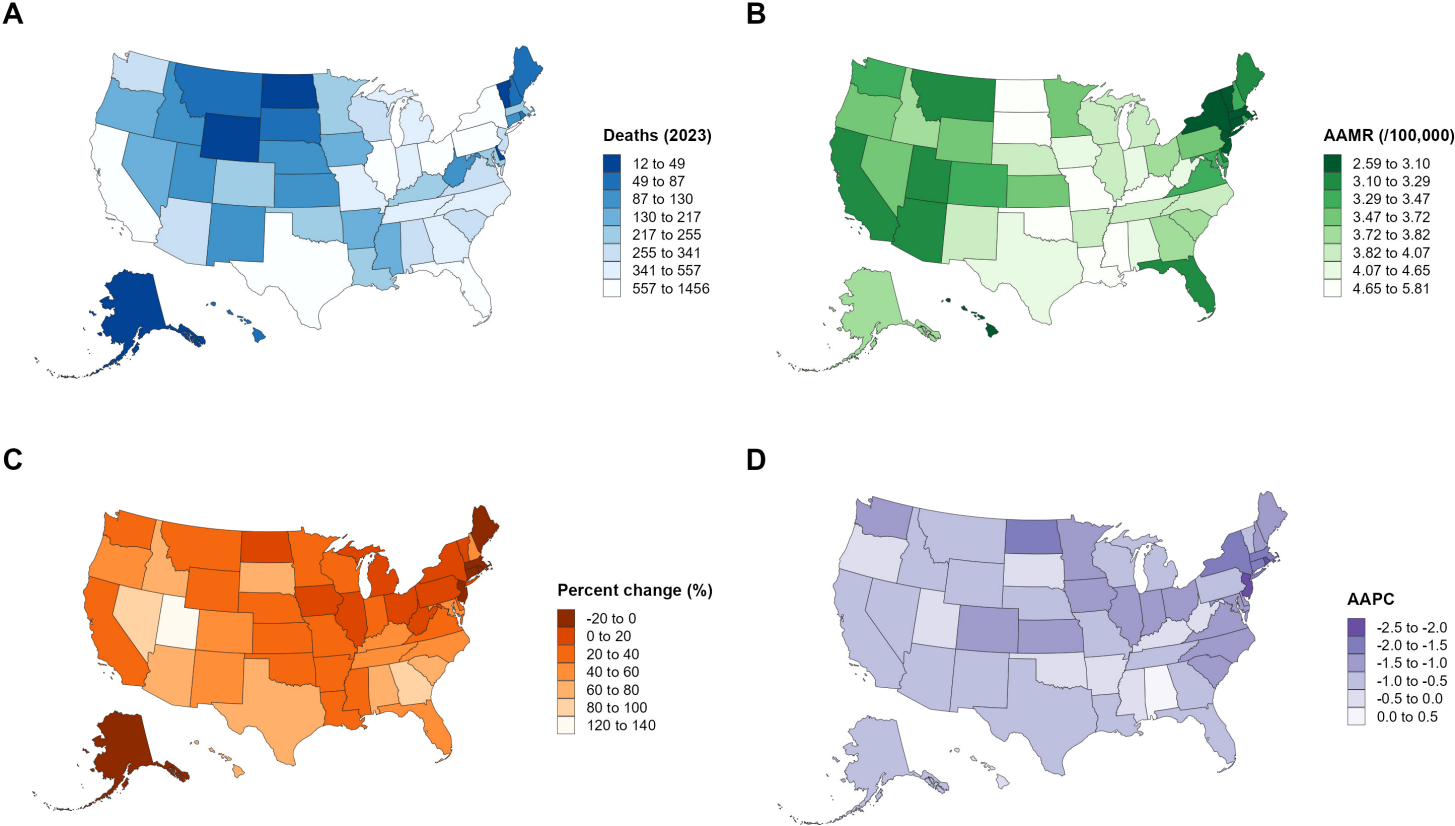
Overall age-adjusted mortality trends of kidney cancer in the United States, 1999–2023.

Between 1999 and 2023, the total number of fatalities due to kidney malignant neoplasm in the United States climbed from 10,912 to 14,549, reflecting a 33.33% surge. Notably, the age-adjusted mortality rate (AAMR) exhibited a significant downward trajectory over the same period: it stood at 4.29 per 100,000 population (95% CI: 4.21–4.37) in 1999 and dropped to 3.61 per 100,000 (95% CI: 3.55–3.67) by 2023, with an average annual percent change (AAPC) of -0.76 (95% CI: -1.10 to -0.41, P < 0.05). Additional analysis via Joinpoint regression uncovered distinct phase-specific patterns in the overall mortality trend: an initial uptick during 1999–2001 (APC: 2.07, 95% CI: -0.64 to 4.85), followed by a steady decrease from 2001 to 2015 (APC: -0.89, 95% CI: -1.02 to -0.76); a more pronounced reduction between 2015 and 2019 (APC: -2.33, 95% CI: -3.52 to -1.12); and a non-statistically significant downward tendency from 2019 to 2023 (APC: -0.12, 95% CI: -0.87 to 0.65) (**Table 1** and **Figure 1**).

**Table 1:** Malignant neoplasm of kidney deaths and AAMR in the United States from 1999 to 2023 and their changing trends.

Characteristic	Deaths			AAMR		
	1999	2023	Percent Change (%)	1999 (95% CI)	2023 (95% CI)	AAPC (95% CI)
Total	10912	14549	33.33	4.29 (4.21 to 4.37)	3.61 (3.55 to 3.67)	-0.76 (-1.10 to -0.41)*
Sex						
Female	4162	4845	16.41	2.84 (2.75 to 2.93)	2.20 (2.14 to 2.27)	-1.27 (-1.53 to -1.02)*
Male	6750	9704	43.76	6.23 (6.08 to 6.38)	5.29 (5.19 to 5.40)	-0.72 (-1.12 to -0.31)*
Census region						
Northeast	2150	2153	0.14	4.04 (3.87 to 4.21)	2.88 (2.76 to 3.01)	-1.55 (-2.13 to -0.97)*
Midwest	2793	3370	20.66	4.64 (4.47 to 4.82)	3.99 (3.85 to 4.12)	-0.70 (-1.48 to 0.09)
South	3892	5970	53.39	4.31 (4.17 to 4.44)	3.90 (3.80 to 4.00)	-0.46 (-1.00 to 0.08)
West	2077	3056	47.14	4.05 (3.88 to 4.23)	3.32 (3.20 to 3.44)	-0.91 (-1.03 to -0.80)*
Race						
Hispanic	540	1536	184.44	3.78 (3.45 to 4.12)	3.51 (3.33 to 3.69)	-0.76 (-1.01 to -0.51)*
NH Black	1014	1383	36.39	4.46 (4.18 to 4.74)	3.45 (3.27 to 3.64)	-1.30 (-1.50 to -1.10)*
NH White	9140	11076	21.18	4.34 (4.25 to 4.43)	3.81 (3.74 to 3.89)	-0.59 (-1.01 to -0.16)*
NH Other	192	12985	6663.02	2.49 (2.11 to 2.86)	3.61 (3.55 to 3.67)	1.22 (-0.96 to 3.45)
Urbanization^1^						
Metropolitan	8727	11399	30.62	4.18 (4.09 to 4.27)	3.49 (3.43 to 3.56)	-1.16 (-1.43 to -0.89)*
Nonmetropolitan	2185	3150	44.16	4.63 (4.43 to 4.82)	4.43 (4.27 to 4.60)	-0.38 (-0.55 to -0.21)*
Age groups^2^						
5–14 years	33	30	-9.09	0.08 (0.06 to 0.11)	0.07 (0.05 to 0.10)	-0.47 (-3.25 to 2.39)
15–24 years	17	31	82.35	0.04 (0.03 to 0.07)	0.07 (0.05 to 0.10)	-1.22 (-2.84 to -0.58)*
25–34 years	54	49	-9.26	0.13 (0.10 to 0.18)	0.11 (0.08 to 0.14)	0.13 (-0.57 to 0.83)
35–44 years	340	225	-33.82	0.75 (0.67 to 0.83)	0.51 (0.44 to 0.57)	-1.81 (-2.22 to -1.41)*
45–54 years	1139	840	-26.25	3.11 (2.93 to 3.29)	2.07 (1.93 to 2.21)	-1.92 (-2.15 to -1.70)*
55–64 years	2085	2565	23.02	8.77 (8.39 to 9.14)	6.13 (5.89 to 6.37)	-1.59 (-2.54 to -0.64)*
65–74 years	3012	4324	43.56	16.35 (15.77 to 16.94)	12.47 (12.09 to 12.84)	-1.35 (-1.92 to -0.78)*
75–84 years	2942	4144	40.86	24.07 (23.20 to 24.94)	22.56 (21.87 to 23.25)	-0.46 (-0.72 to -0.19)*
85+ years	1290	2341	81.47	31.05 (29.36 to 32.75)	37.79 (36.26 to 39.32)	0.58 (0.16 to 1.00)*

*P<0.05. ^1^In the context of urbanization, the 2023 AAMR data was substituted with that from 2020, and the AAPC was calculated based on the period from 1999 to 2020. ^2^For the age groups, the crude mortality rate was used as a substitute for AAMR, and the AAPC was computed based on the crude mortality rate. AAMR, age-adjusted mortality rate; CI, confidence interval; AAPC, average annual percent change; NH, non-Hispanic.

### 3.2 Mortality by sex

**Figure 2:**
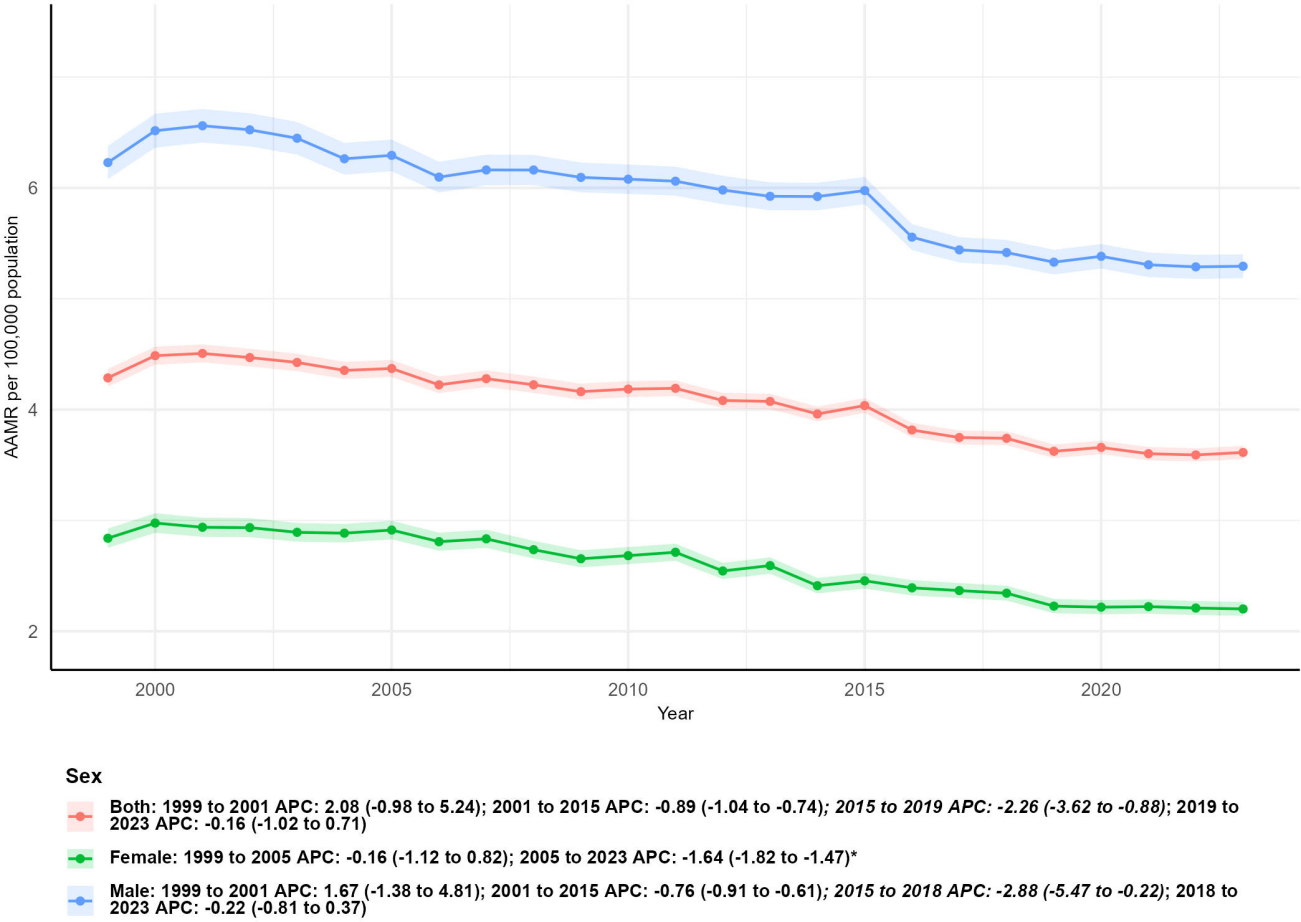
Age-adjusted mortality trends of kidney cancer by sex, 1999–2023.

Notable sex-based disparities in kidney malignant neoplasm mortality were evident throughout the 1999–2023 study period. The total number of deaths among males surged from 6,750 in 1999 to 9,704 in 2023, marking a substantial 43.76% increase, whereas female deaths rose moderately from 4,162 to 4,845, corresponding to a 16.41% uptick. Consistently, age-adjusted mortality rates (AAMRs) for males remained higher than those for females in both anchor years: in 1999, the male AAMR was 6.23 per 100,000 population (95% CI: 6.08–6.38), compared to 2.84 per 100,000 (95% CI: 2.75–2.93) for females; by 2023, these rates had declined to 5.29 per 100,000 (95% CI: 5.19–5.40) for males and 2.20 per 100,000 (95% CI: 2.14–2.27) for females.Both sexes exhibited statistically significant downward trends in AAMR over the entire period (females: AAPC = -1.27, 95% CI: -1.53 to -1.02, P < 0.05; males: AAPC = -0.72, 95% CI: -1.12 to -0.31, P < 0.05). A formal pairwise comparison of the AAPCs revealed a 95% confidence interval for the difference ranging from -1.01 to -0.11, indicating that the reduction in AAMR was more prominent among females than males.Temporal mortality patterns also diverged by sex. For males, the trend was characterized by an initial slight uptick from 1999 to 2001 (APC: 1.67, 95% CI: -1.38 to 4.81), followed by a steady decline during 2001–2015 (APC: -0.76, 95% CI: -0.91 to -0.61), a more pronounced reduction between 2015 and 2018 (APC: -2.88, 95% CI: -5.47 to -0.22), and a non-statistically significant mild decrease from 2018 to 2023 (APC: -0.22, 95% CI: -0.81 to 0.37). In contrast, females experienced stable mortality rates from 1999 to 2005 (APC: -0.16, 95% CI: -1.12 to 0.82), followed by a consistent and significant downward trajectory from 2005 to 2023 (APC: -1.64, 95% CI: -1.82 to -1.47, P < 0.05) (**Table 1**; **Figure 2**).

### 3.3 Mortality by race/ethnicity

Striking disparities in kidney malignant neoplasm mortality were identified across different racial and ethnic groups during the 1999–2023 study period. The most dramatic surges in total deaths were observed among Hispanic individuals (climbing from 538 to 1,531, a 184.57% jump) and non-Hispanic Other populations (rising from 192 to 528, an approximate 175.00% increase). Subsequently, non-Hispanic Black individuals saw a mortality increase from 1,005 to 1,364, corresponding to a 35.72% uptick, while non-Hispanic White individuals experienced the most modest growth, with deaths increasing from 9,142 to 1,1083 (a 21.23% rise).

In 1999, non-Hispanic Black individuals had the highest age-adjusted mortality rate (AAMR) at 4.43 per 100,000 population (95% CI: 4.14–4.72), whereas non-Hispanic Other populations had the lowest rate at 2.47 per 100,000 (95% CI: 2.10–2.84). By 2023, AAMRs had decreased across all racial and ethnic subgroups: non-Hispanic Black individuals maintained a relatively higher AAMR of 3.38 per 100,000 (95% CI: 3.20–3.56), and non-Hispanic Other populations continued to have the lowest rate at 1.80 per 100,000 (95% CI: 1.65–1.95).

All racial and ethnic groups demonstrated statistically significant downward trends in AAMR over the entire study period: non-Hispanic Black (AAPC = -1.32, 95% CI: -1.52 to -1.12, P < 0.05), Hispanic (AAPC = -0.75, 95% CI: -1.00 to -0.50, P < 0.05), non-Hispanic White (AAPC = -0.58, 95% CI: -1.00 to -0.16, P < 0.05), and non-Hispanic Other (AAPC = -1.00, 95% CI: -1.80 to -0.20, P < 0.05).

**Figure 3:**
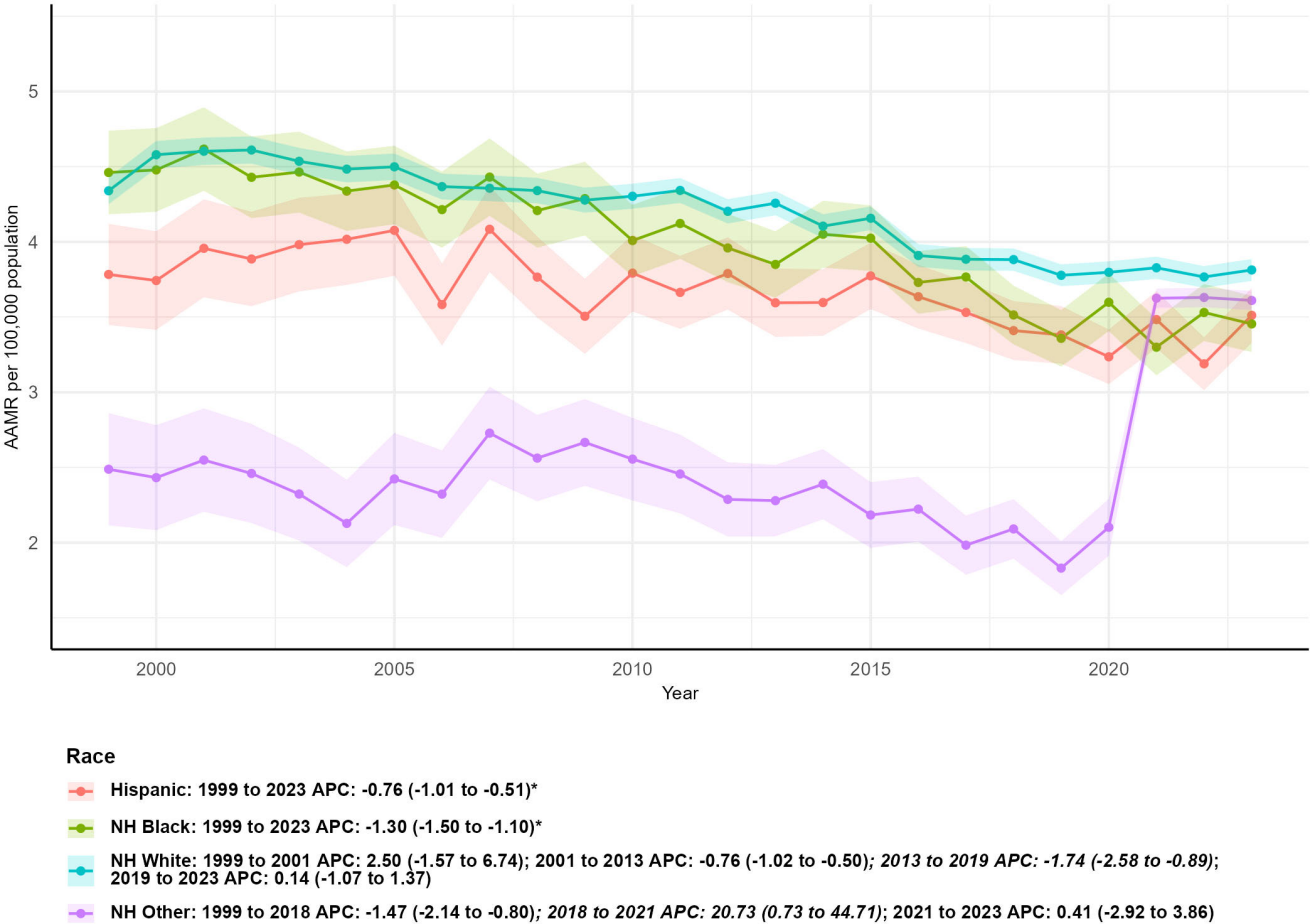
Age-adjusted mortality trends of kidney cancer by race/ethnicity, 1999–2023.

Temporal mortality patterns varied substantially among groups. Non-Hispanic White individuals experienced an initial uptick in mortality from 1999 to 2001 (APC = 2.41, 95% CI: -1.60 to 6.59), followed by consecutive declines from 2001 to 2013 (APC = -0.74, 95% CI: -0.99 to -0.48) and 2013 to 2019 (APC = -1.71, 95% CI: -2.54 to -0.88), and then stable mortality rates with no significant change from 2019 to 2023 (APC = 0.08, 95% CI: -1.11 to 1.29). Non-Hispanic Other populations maintained stable mortality from 1999 to 2009 (APC = 0.70, 95% CI: -1.06 to 2.48), followed by a marked decline from 2009 to 2023 (APC = -2.21, 95% CI: -2.98 to -1.44, P < 0.05). In contrast, both Hispanic and non-Hispanic Black populations showed consistent downward mortality trends throughout the entire 1999–2023 period (**Table 1**; **Figure 3**).

### 3.4 Mortality by census region

**Figure 4:**
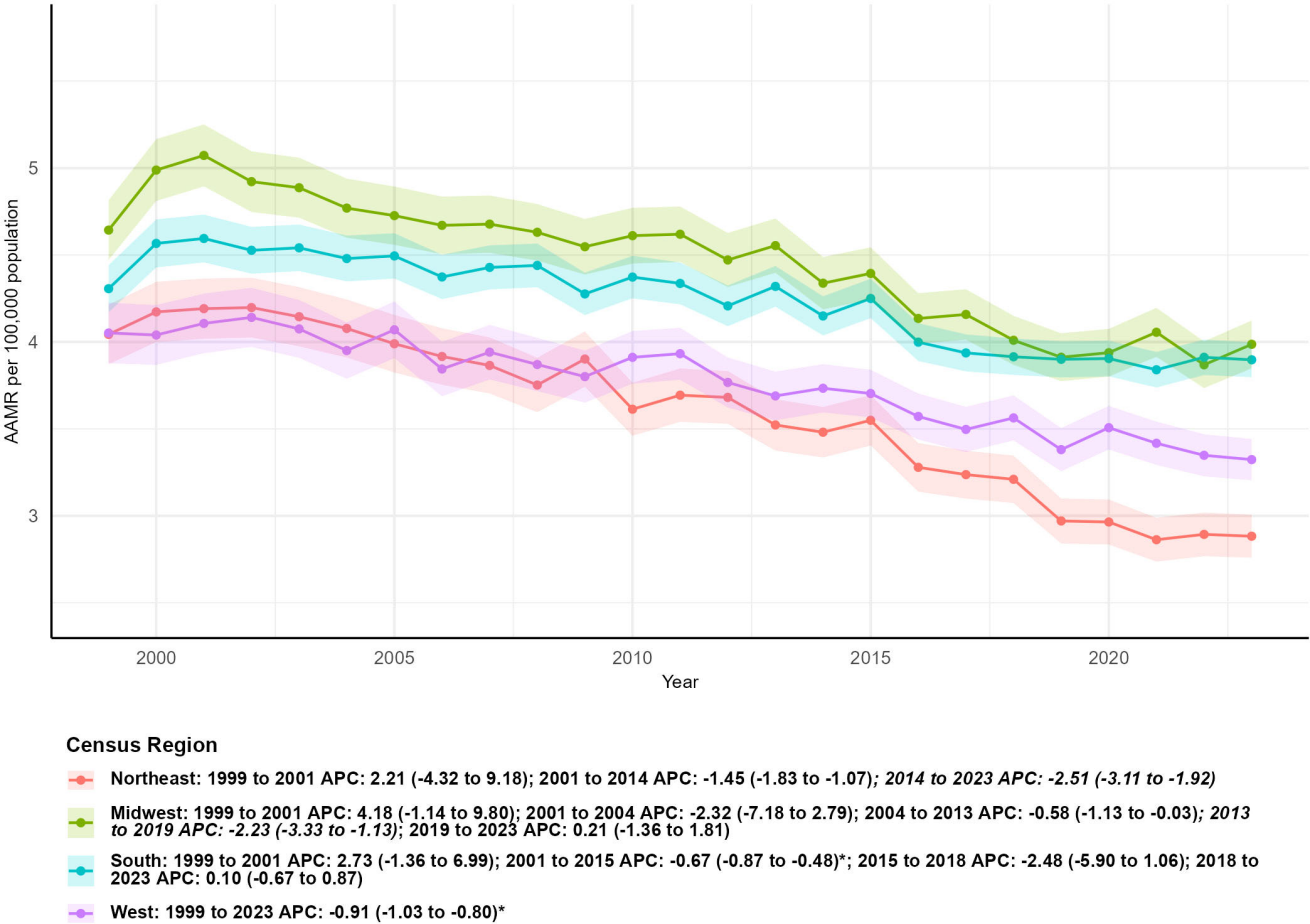
Age-adjusted mortality trends of kidney cancer by U.S. census region, 1999–2023.

Notable geographic disparities in kidney malignant neoplasm mortality trends were identified across U.S. Census Regions during the 1999–2023 study period. The Southern region consistently had the highest number of deaths, rising from 3,892 in 1999 to 5,963 in 2023—marking the most substantial growth of 53.21%. This was followed by the Western region, where fatalities increased from 2,075 to 3,058 (a 47.38% surge); the Midwest, with deaths climbing from 2,791 to 3,372 (a 20.82% uptick); and the Northeast, which saw a minimal rise from 2,144 to 2,155 (a mere 0.51% increase).In 1999, the Midwest reported the highest age-adjusted mortality rate (AAMR) at 4.60 per 100,000 population (95% CI: 4.42–4.78), while the Northeast and West had comparable rates of 4.03 and 4.04 per 100,000, respectively. By 2023, the Northeast achieved the lowest AAMR at 2.88 per 100,000 (95% CI: 2.76–3.00), whereas the South emerged as the region with the highest AAMR at 3.85 per 100,000 (95% CI: 3.75–3.96).Statistically significant declines in AAMR were observed in three regions: the Northeast (AAPC = -1.55, 95% CI: -2.16 to -0.94, P < 0.05), the South (AAPC = -0.53, 95% CI: -1.00 to -0.05, P < 0.05), and the West (AAPC = -0.90, 95% CI: -1.01 to -0.79, P < 0.05). In contrast, the Midwest exhibited a non-statistically significant downward trend (AAPC = -0.69, 95% CI: -1.47 to 0.10). Temporal mortality patterns differed across regions. The Northeast experienced an initial non-significant rise from 1999 to 2001 (APC = 2.06, 95% CI: -4.79 to 9.40), followed by consistent declines from 2001 to 2014 (APC = -1.40, 95% CI: -1.80 to -1.00) and a more pronounced reduction from 2014 to 2023 (APC = -2.55, 95% CI: -3.18 to -1.93, P < 0.05). The Midwest showed variable trends, including a non-statistically significant slight increase from 2019 to 2023 (APC = 0.16, 95% CI: -1.40 to 1.74). Both the South and West maintained steady downward mortality trends across most subperiods, aligning with broader national patterns of reduced kidney cancer mortality in metropolitan areas (**Table 1**; **Figure 4**).

### 3.5 Mortality by level of urbanization

**Figure 5:**
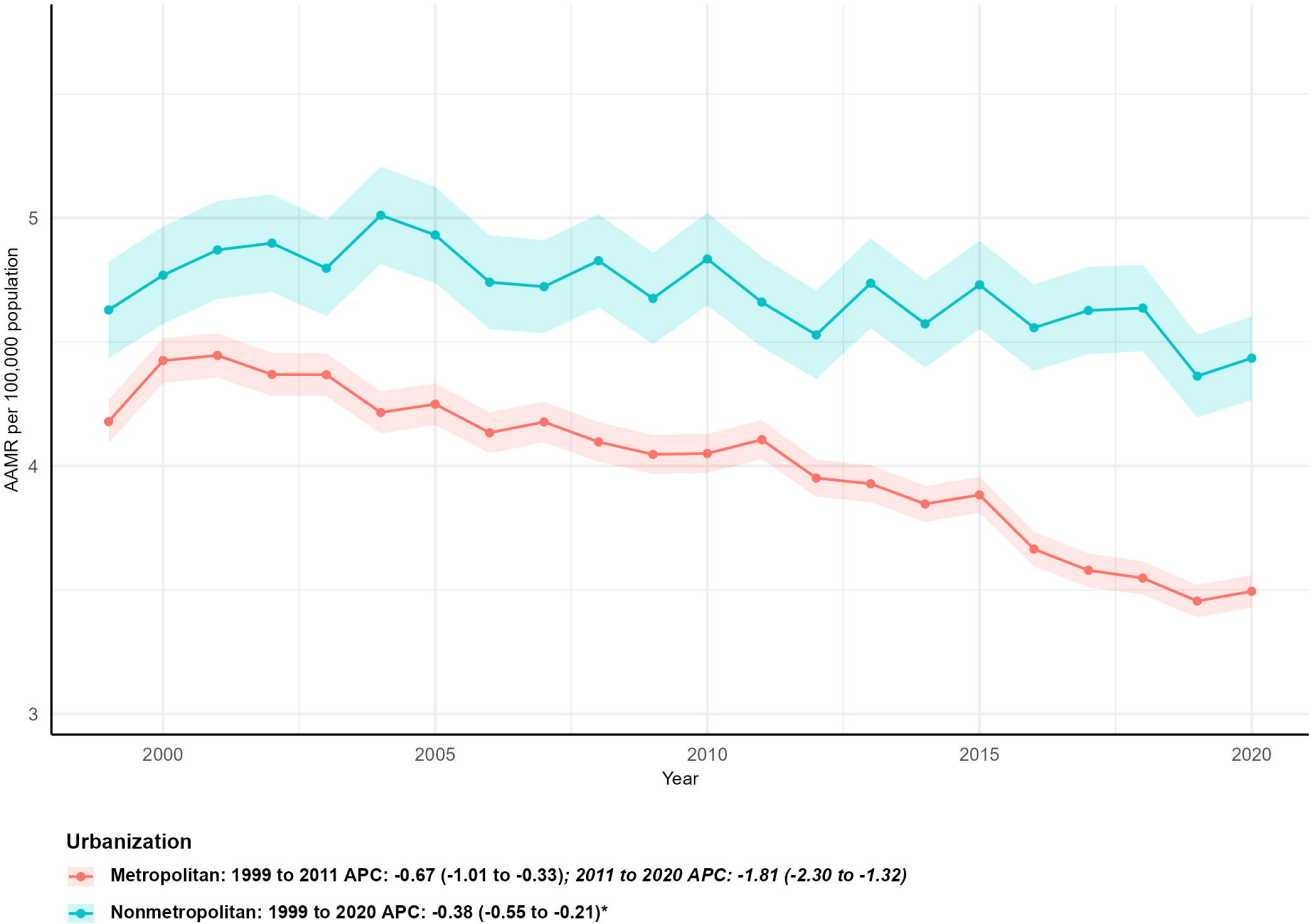
Age-adjusted mortality trends of kidney cancer by urbanization level, 1999–2023.

Clear disparities in kidney malignant neoplasm mortality between metropolitan and nonmetropolitan areas persisted throughout the 1999–2023 study period. Metropolitan areas accounted for a larger total number of deaths, with figures climbing from 8,703 in 1999 to 11,382 in 2023—a 30.78% increase. However, this growth rate was lower than that of nonmetropolitan areas, where deaths rose from 2,190 to 3,147, corresponding to a 43.70% surge. In 1999, nonmetropolitan areas had a higher age-adjusted mortality rate (AAMR) of 4.63 per 100,000 population (95% CI: 4.43–4.83), compared to 4.17 per 100,000 (95% CI: 4.08–4.26) in metropolitan areas. This pattern remained consistent into 2023: nonmetropolitan areas maintained an AAMR of 4.43 per 100,000 (95% CI: 4.26–4.60), while metropolitan areas recorded a lower AAMR of 3.47 per 100,000 (95% CI: 3.40–3.54). Both urbanization levels exhibited statistically significant reductions in AAMR: metropolitan areas had an AAPC of -1.19 (95% CI: -1.45 to -0.93, P < 0.05), and nonmetropolitan areas had an AAPC of -0.38 (95% CI: -0.55 to -0.21, P < 0.05). Notably, metropolitan areas showed two distinct phases of decline: an initial moderate drop from 1999 to 2012 (APC = -0.71, 95% CI: -1.00 to -0.42, P < 0.05), followed by a more pronounced reduction from 2012 to 2020 (APC = -1.96, 95% CI: -2.52 to -1.39, P < 0.05) (**Table 1**; **Figure 5**).

### 3.6 Results by age group

**Figure 6:**
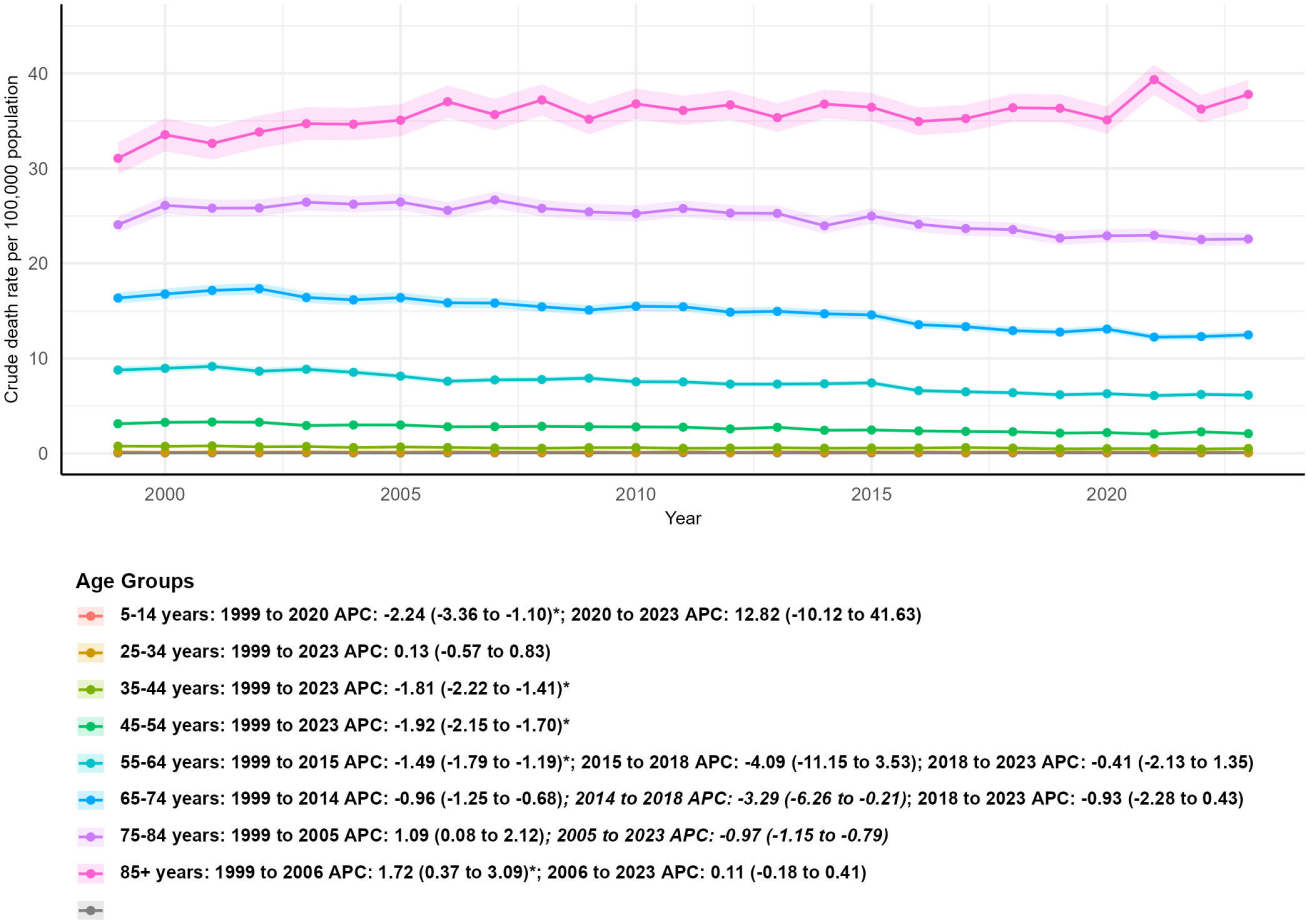
Crude mortality trends of kidney cancer by age group, 1999–2023.

Age-specific mortality was evaluated using CMRs because age adjustment within predefined age groups is not epidemiologically valid. Notably, increases in CMR among elderly subgroups reflect population aging and growth rather than elevated disease-specific mortality risk. Individuals aged 5–14 years exhibited very low and stable CMRs, decreasing slightly from 0.08 per 100,000 in 1999 to 0.07 per 100,000 in 2023, representing a 9.09% reduction with a non-significant AAPC of −0.47 (95% CI: −3.25 to 2.39, P>0.05). Individuals aged 15–24 years showed an 82.35% increase in CMR from 0.04 to 0.07 per 100,000, likely driven by small case numbers and demographic shifts, despite a statistically significant AAPC of −1.22 (95% CI: −2.84 to −0.58, P<0.05). All young and middle-aged adult groups aged 25–64 years demonstrated significant reductions in CMR, with AAPCs ranging from −1.81 to −1.59 (all P<0.05). Among adults aged 65–74 and 75–84 years, CMRs increased by 43.56% and 40.86%, respectively, driven by population aging, yet both subgroups exhibited statistically significant declines in age-specific mortality risk as reflected by negative AAPCs of −1.35 and −0.46 (both P<0.05). Individuals aged 85 years and older had the highest CMR, which increased markedly from 31.05 to 37.79 per 100,000 (an 81.47% rise), with a significant positive AAPC of 0.58 (95% CI: 0.16 to 1.00, P<0.05); this trend may be attributable to population aging, reduced screening utilization, higher comorbidity burden, and increased frailty in the oldest old (**Table 1**; **Figure 6**).

## 4 Discussion

This retrospective, population-based investigation comprehensively examined the temporal dynamics and sociodemographic inequities in mortality associated with malignant neoplasm of the kidney across the United States over a quarter-century (1999–2023). A defining observation was that while the absolute count of kidney cancer-related deaths surged from 10,912 to 14,549—largely driven by the nation’s expanding and aging population—the age-adjusted mortality rate (AAMR), a more robust metric that accounts for population age structure, declined markedly from 4.29 to 3.61 per 100,000 individuals. This divergent pattern underscores the dominant impact of population aging on absolute mortality counts, rather than worsening disease-specific risk, while persistent disparities across sex, race/ethnicity, geographic region, urbanization level, and age strata highlight the evolving landscape of kidney cancer burden and the need for targeted public health interventions (10). The downward AAMR trend coincides with improvements in diagnostic, surgical, and systemic therapies for kidney cancer, although definitive causal conclusions cannot be drawn in the absence of individual-level clinical data (11).

The rise in absolute kidney cancer deaths aligns with two intertwined demographic and clinical drivers. First, the ongoing aging of the U.S. population has naturally inflated mortality counts, as kidney cancer risk escalates dramatically with advancing age. Second, refinements in diagnostic imaging technologies, including computed tomography (CT) and magnetic resonance imaging (MRI), have boosted the detection of incidental kidney tumors, ensuring that previously unrecognized cases are now accurately identified (10). The observed reduction in AAMR corresponds temporally with the broader adoption of minimally invasive surgery, targeted agents, and immune checkpoint inhibitors, but these associations do not confirm causation given the ecological study design (11). The phase-specific AAMR trends—an initial modest uptick (1999–2001), followed by a steady decline (2001–2015), a steeper reduction (2015–2019), and recent stabilization (2019–2023)—parallel the timeline of therapeutic advances, yet firm causal attribution is limited by the lack of clinical and treatment information in the database.

Sex-based differences persisted throughout the study period, with males experiencing higher mortality counts and rates than females—findings consistent with prior research linking male sex to elevated kidney cancer risk (12, 13). This pattern may be related to higher exposure to modifiable risk factors such as tobacco use, alcohol consumption, and obesity, as well as inherent biological variations including hormone profiles and renal physiology (14, 15). The more pronounced AAMR decline among females may be associated with differences in health-seeking behavior, comorbidity burden, or care access, but these mechanisms cannot be verified in this dataset.

Despite overall mortality improvements, racial and ethnic disparities remain a pressing public health challenge (16, 17). While all racial/ethnic groups demonstrated significant AAMR reductions, Hispanic and non-Hispanic (NH) Other populations saw the largest proportional increases in death counts—likely driven by rapid population growth. NH Black individuals maintained the highest AAMRs in both 1999 and 2023; this pattern may be associated with socioeconomic barriers, limited insurance coverage, higher prevalence of hypertension and diabetes, and potential genetic susceptibilities (18, 19). These observations highlight persistent inequities but do not establish causal pathways due to missing socioeconomic and clinical covariates.

Regional variations in mortality underscore the role of healthcare access and population health profiles (20–24). The South region recorded the highest number of deaths and the highest 2023 AAMR, which may coincide with higher rates of obesity, hypertension, and tobacco use, as well as uneven access to specialized oncologic care (20–22). In contrast, the Northeast exhibited the steepest AAMR decline and the lowest 2023 rate, which may overlap with stronger healthcare infrastructure and earlier adoption of evidence-based treatments (23, 24). These geographic patterns are descriptive and do not imply direct causality.

Urban-rural disparities were particularly noteworthy: nonmetropolitan areas consistently had higher AAMRs than metropolitan areas. Rural populations face well-documented obstacles including shortages of urologic and oncologic providers, long travel distances to tertiary centers, and lower insurance coverage, which may contribute to delayed diagnosis and limited treatment access (25–27). The slower mortality reduction in rural areas is consistent with these structural barriers, but causal links cannot be confirmed in this ecological analysis.

Age emerged as a pivotal determinant of mortality trajectories. Children and adolescents (5–24 years) exhibited extremely low crude mortality rates, reflecting the rarity of kidney cancer in these groups, with cases often linked to genetic syndromes or rare histologic subtypes (28). Young and middle-aged groups (25–64 years) demonstrated consistent mortality reductions, consistent with earlier detection and improved access to routine care. In contrast, the 85+ years group had the highest and increasing crude mortality rate, which may be related to reduced screening utilization, higher comorbidity burden, greater frailty, and less aggressive treatment paradigms in the oldest old (29–31). These age-related patterns are descriptive and do not establish causal drivers.

### 4.1 Strengths and limitations

This study has several strengths. Firstly, the use of the CDC-WONDER database ensures access to population-level, nationally representative mortality rates, enabling robust analysis of long-term trends across multiple sociodemographic strata. The application of Joinpoint regression provides detailed insights into changes during specific time periods, and the inclusion of AAMR adjusts for age-related bias in mortality rate comparisons.

Several limitations must be emphasized in accordance with STROBE guidelines. First, the database lacks clinical details including tumor stage, histologic subtype, treatment modality, and comorbidity profile, which restricts causal interpretation of observed trends. Second, misclassification of the underlying cause of death may occur, particularly among older adults with multiple chronic conditions, introducing potential residual bias. Third, the study does not capture socioeconomic factors including income, education, and insurance status, which are key mediators of cancer disparities. Fourth, the ecological study design limits individual-level inference, and residual confounding cannot be fully excluded.

## 5 Conclusion

In conclusion, age-adjusted mortality from malignant neoplasm of the kidney declined significantly in the United States between 1999 and 2023, while absolute mortality counts increased due to population aging and growth. Divergent trends between crude and age-adjusted mortality rates emphasize the critical importance of appropriate statistical adjustment in interpreting population-level cancer statistics. Substantial and persistent disparities exist across sex, race/ethnicity, geographic region, urbanization level, and age, highlighting the need for targeted, equitable public health interventions. Future efforts should prioritize expanded access to screening, early detection, high-quality specialized care, and culturally appropriate health education in underserved regions and vulnerable populations to reduce disparities and improve outcomes for all individuals affected by kidney cancer.

## Data Availability

The datasets generated and/or analyzed during the current study are available in the CDC wonder database (https://wonder.cdc.gov/mcd-icd10.html).
